# Emerging insights and challenges for understanding T cell function through the proteome

**DOI:** 10.3389/fimmu.2022.1028366

**Published:** 2022-11-16

**Authors:** Laura A. Solt

**Affiliations:** ^1^ Department of Immunology and Microbiology, University of Florida (UF) Scripps Biomedical Research, Jupiter, FL, United States; ^2^ Department of Molecular Medicine, University of Florida (UF) Scripps Biomedical Research, Jupiter, FL, United States

**Keywords:** adaptive immunity, T cells, mass spectrometry, proteomics, protein identification

## Abstract

T cells rapidly transition from a quiescent state into active proliferation and effector function upon exposure to cognate antigen. These processes are tightly controlled by signal transduction pathways that influence changes in chromatin remodeling, gene transcription, and metabolism, all of which collectively drive specific T cell memory or effector cell development. Dysregulation of any of these events can mediate disease and the past several years has shown unprecedented novel approaches to understand these events, down to the single-cell level. The massive explosion of sequencing approaches to assess the genome and transcriptome at the single cell level has transformed our understanding of T cell activation, developmental potential, and effector function under normal and various disease states. Despite these advances, there remains a significant dearth of information regarding how these events are translated to the protein level. For example, resolution of protein isoforms and/or specific post-translational modifications mediating T cell function remains obscure. The application of proteomics can change that, enabling significant insights into molecular mechanisms that regulate T cell function. However, unlike genomic approaches that have enabled exquisite visualization of T cell dynamics at the mRNA and chromatin level, proteomic approaches, including those at the single-cell level, has significantly lagged. In this review, we describe recent studies that have enabled a better understanding of how protein synthesis and degradation change during T cell activation and acquisition of effector function. We also highlight technical advances and how these could be applied to T cell biology. Finally, we discuss future needs to expand upon our current knowledge of T cell proteomes and disease.

## Introduction

An effective immune response involves the clonal expansion of antigen specific T cells into effector cells, which is fundamental to adaptive immunity. The programs that underlie this response require the combined actions of multiple signaling pathways that activate metabolic pathways and transcription factors to initiate a series of events leading to massive changes in cell size, proliferation, and differentiation. Activated T cells can, at a minimum, double in size over the course of 1-2 days (1). Following this change, T cells undergo rapid proliferation, doubling every 6-12 hours (2). Further, depending on the environmental milieu, antigen specific CD4^+^ T cells can differentiate into any number of T helper lineages (i.e., T_H_1, T_H_2, T_H_17, etc.). Alternatively, antigen specific CD8^+^ T cells can expand and develop into “effector” and “memory” populations, acquiring differences in their life spans and effector functions as infection persists or is cleared. Advances in genomic and transcriptomic techniques have significantly advanced our understanding of the transcriptional regulation of T cells during immune responses, in some instances, down to the single cell level (3). While there is clearly more to be done on those fronts, central to all these processes are protein synthesis. Proteins are the building blocks of the cell and fundamental to the execution of the genetic code. Protein expression is often inferred from mRNA expression, but these correlations are not always accurate, nor do they account for post-translational modifications (PTM), cellular localization, cleavage events, or various isoforms (4–6). Thus, our basic understanding of the T cell proteome during immune responses is considerably lacking when compared to the recent advances in genomics and transcriptomics. Importantly, subtle changes in protein structure, folding, or PTM status can have significant consequences to our health, changes that may not be observed at the mRNA level. Therefore, proteins can be the cause (i.e., Alzheimer’s disease) or the cure (i.e., use of antibodies as therapeutics) for disease. In this review, we describe our current toolbox of techniques to study proteins in T cells, the importance of proteomics, and recent studies that have given us a better understanding of how the protein landscape changes during T cell activation and acquisition of effector function. We also discuss technical advances in proteomics and future needs to expand upon our current knowledge of T cell proteomes and make this a more mainstream application for T cell biology.

## An immunologist’s current toolbox to measure proteins

Immunologists utilize several techniques to analyze proteins in T cells, some down to the single cell level. Protein profiling techniques include, but are not limited to, enzyme-linked immunosorbent assays (ELISA), immunohistochemistry, western blotting, flow cytometry-based techniques, and cellular indexing of transcriptomes and epitopes by sequencing (CITE-seq). ELISA’s, immunohistochemistry, and western blotting are low-throughput techniques and rely on the availability of specific antibodies, but antibodies are expensive and limited by the number of antigens they probe. ELISA’s can probe one factor at a time or be multiplexed (i.e., Luminex technology), but these assays can be costly. Single-cell western blotting (scWesterns) applications were introduced several years ago and enabled the measure of cell-to-cell heterogeneity on a microscope slide (7). However, this method is also low throughput and limited to the number of antibodies available to your protein(s) of interest.

Several flow cytometry-based techniques have evolved to enable quantification of proteins at the single-cell level. Traditional flow cytometry, also referred to as polychromatic cytometry, relies on a series of bandpass filters to isolate a small spectral window that favors the specific fluorophore being analyzed. Up to 30 subsets can be defined based on this method (8). The introduction of spectral flow cytometry has expanded upon the limitations of traditional, polychromatic flow cytometry to enable visualization of up to 40 parameters on immune cells (8). Cytometry time of flight (CyTOF) is a variation of flow cytometry in which antibodies are labeled with heavy metal ion tags in lieu of fluorophores (9). CyTOF is a powerful tool to quantify labeled targets with simultaneous visualization of up to 60 individual parameters at once and is particularly useful for in-depth analysis of immune populations with limited samples. CyTOF was particularly useful in diversifying subsets of T cell exhaustion states from patients with HIV and cancer (10). CyTOF was also used in conjunction with metabolic assays to define the metabolic states of human naïve and memory cytotoxic T cells to establish a more robust understanding of functional states in T cells (11). Used in conjunction with other approaches, CyTOF as well as other flow cytometry-based techniques have given us significant mechanistic insight into T cell biology both in normal and disease states. However, much like techniques outlined above, these methods are limited to availability of antibodies to your protein(s) of interest and number of parameters that can be analyzed per sample.

CITE-seq is a more recently described method to perform concurrent measurements of both RNA and protein transcripts at the single cell level. CITE-seq uses a combination of oligonucleotide-labeled antibodies to integrate protein profiling with single-cell droplet RNA-sequencing to collate RNA levels and amount of antibody present (as a proxy for protein levels) per cell. Multiple antibodies can be used per cell to gain qualitative and quantitative information on surface proteins. This method was recently used to characterize age-associated alterations in immune cells. Using this method, the authors of this study identified a sub-population of age-associated CD8^+^ T cells that had hallmarks of T cell exhaustion and tissue homing (12), suggesting these cells could be a potential target for age-associated immune dysfunction. Another study used a combination of CITE-seq with TCR sequencing to identify a neoantigen-reactive T cell signature for CD4^+^ and CD8^+^ T cells in non-small cell carcinoma (13). This approach could enable rapid identification of neoantigen-reactive TCRs and expedite personalized medicine through engineering of patient, neoantigen-specific T cells. Finally, using a combination of CyTOF, CITE-seq, and scRNA-seq to resolve immune diversity in atherosclerotic plaques from patients with cardiovascular disease (CVD), authors from this study uncovered significant diversity in the CD4^+^ T cell subsets. The authors identified subsets of T cells that were activated, some differentiated, whereas some expressed markers of exhaustion (14). Data generated from these techniques may enable the design of more precise therapeutics for CVD. These approaches are extremely useful, particularly when used in combination. While they clearly have major advantages over the use of single-cell RNA-seq alone, similar to flow cytometry-based approaches, CITE-seq is limited by the number of markers that can be used for cellular detection. These approaches are incredibly powerful systems to interrogate T cell functionality, but they do not provide a holistic overview of protein expression or changes within a cell population in response to various stimuli. Other approaches need to be implemented for this visualization.

## Proteomics

In the study of biological systems, we have genomics, transcriptomics, and proteomics. In its most rudimentary form, proteomics is the study of proteins, specifically, how different proteins interact with each other. Multiple subcategories of proteomics exist with the purpose to broaden our understanding of protein composition within a cell or target tissue: expression proteomics – the quantitative study of proteins between samples; functional proteomics - the assessment of protein-protein interactions elucidating biological function; structural proteomics – determination of three-dimensional protein structures on a genome-wide scale; chemoproteomics – assessment of protein-small molecule interactions. While there are multiple techniques that can measure protein/protein interactions in cells, mass spectrometry (mass-spec, MS) is one that is central to these applications. It is a comprehensive tool capable of not only identifying proteins, but also characterizing protein structure, protein isoforms, protein post-translational modifications (PTMs), and protein-protein interactions both quantitatively and qualitatively. Mass-spec is also a powerful tool to identify lipid species and metabolites. While all subcategories and species identification are worth in-depth discussion, for the purposes of this review, we focus on expression proteomics and protein identification.

Mass spectrometry largely uses two major workflow approaches to identify proteins: a bottom-up approach, also called “shotgun” proteomics, and a top-down approach. The conventional bottom-up proteomics involves enzymatic trypsin digestion of proteins into peptides suited for separation by high-performance liquid chromatography (HPLC), ionization, and fragmentation-based sequencing in the tandem mass spectrometer. Proteins are digested into peptides of 5-20 amino acids in length (15). Alternatively, liquid chromatography-MS (LC-MS) can be used if samples have been treated with chemicals to separate compounds before ionization which is conveyed to the mass-spectrometer (16). Bottom-up proteomics has become a routine procedure due to high performance instrumentation, improvements in enrichment tools, availability of high-resolution mass-spectrometers, and innovative data acquisition methods. However, since protein is digested into small peptides, sensitivity can be compromised (17, 18). For example, the ability to map PTMs, truncations, mutations, and polymorphisms in individual proteins is limited by the analysis of the peptides, often leaving gaps and blurring context (19). Peptide masses are correlated with known databases using search engines, including Mascot or Sequest, and if a database is incomplete or amino acids have identical masses (i.e., leucine and isoleucine), only a limited number of fragments may be identified.

Top-down proteomics is a method to characterize and identify intact proteins. This method presents with distinct advantages as well as its own challenges. The most compelling advantage is that it has the potential to quantify intact protein and its modifications, including PTMs. However, due to limitations in instrumentation, this approach is approximately 100-fold less sensitive than bottom-up MS (15) and is limited to proteins on average of 50kDa due to difficulties producing gas-phase fragmentation of proteins (15). Additionally, those proteins of low abundance, including transcription factors, may also be missed using this method. Nevertheless, this technique offers the potential to map protein sequences and localize multiple PTMs on each protein, providing a comprehensive overview of the protein in the cell (19). Using both bottom-up and top-down-MS, recent proteomic studies have provided unprecedented insights into both human and mouse T cell function.

## Applying proteomics to study T cell function

Transcriptional profiling and downstream network analysis has been instrumental to our understanding of T cell function in the immune system. However, T cell activation is dynamic inducing a series of signaling events that cannot be captured by transcriptomics alone. These events include rapid protein translation, PTMs, and changes in cellular metabolism, all of which are key regulators of T cell activation and function. Recent advances in mass-spectrometry, using techniques such as tandem mass tag (TMT), has enabled sample multiplexing, which supports identification and quantification of proteins from multiple samples or tissues. Using this approach, one group set out to capture key T cell activation events by activating naïve mouse T cells with anti-CD3 and anti-CD28 for 2, 8, and 16h, and subjected these cells to both whole proteome analysis and phosphoproteome profiling (20). Consistent with their initial hypothesis, there was weak correlation between the proteome and transcriptome induced by TCR signaling with mRNA expression significantly lagging behind changes in protein expression. TCR signaling led to distinct phases of activation. Initial TCR activation led to a rapid induction of phosphorylation events (within 2h) with limited changes in overall protein abundance (early phase). Between 8-16h (late phase), extensive reprogramming of the proteome and phosphoproteome occurred, leading to changes in protein translation and mitochondrial/metabolic functions. Application of a comprehensive analysis pipeline led to predictions about naïve T cell exit from quiescence into activation, including downregulation of DNA-damage responses and MAPK signaling pathways. This study identified molecular circuitry involved in the exit from T cell quiescence with important implications in T cell maintenance and survival. Importantly, this T cell circuitry could not have been established without global proteomic and phosphoproteomic analysis.

PTMs clearly play a significant role regulating T cell activation and downstream responses. However, there are a number of PTMs outside of phosphorylation that can influence T cell activation, including ubiquitination. Ubiquitin can regulate protein abundance and activity following TCR-CD28 engagement (21–23). Ubiquitin is covalently attached to lysine residues and is often associated with proteasomal or lysosomal degradation and most reports associate ubiquitin-mediated degradation with regulation of T-cell activation (24). However, increasing evidence indicates non-degradative outcomes of ubiquitylation influenced T cell signaling responses, including ubiquitylation of the p85 subunit of PI3K which impacts its recruitment to CD28 and TCRζ (25). Therefore, one group performed proteomics and transcriptomics in primary mouse CD4^+^ T cell to establish degradative vs. non-degradative outcomes of ubiquitylation (26). This group identified 5,500 proteins in primary CD4^+^ T cells. Similar to the findings by Tan et al., TCR-induced transcriptional changes correlated poorly with protein abundance (20, 26). To interrogate ubiquitylation events, they designed an approach using di-glycine remnant profiling. Di-glycine remnants results from cleavage events within ubiquitin when attached to its lysine substrate. This generates a ubiquitin remnant that can be enriched with antibodies and subjected to mass spectrometry. Using this approach, approximately 1,200 substrates of ubiquitylation in CD4^+^ T cells was identified helping to determine that T cell activation drives both degradative and non-degradative ubiquitylation. Proteins undergoing ubiquitin-mediated degradation events included NFKB1, ZAP-70, and LAT. In contrast, proteins that were ubiquitylated but not degraded included CD3ϵ, CD3γ, CDC42, RAC1, RHOA, and GRAP. Combining whole cell proteomics, di-glycine remnant profiling, and transcriptomics, this group established a framework to predict TCR-stimulation dependent ubiquitylation events in rested vs. stimulated CD4^+^ T cells. This study provides a valuable resource to probe non-degradative TCR-dependent ubiquitylation events and their effects on T cell activation and function, a resource that would not be available with genomic and transcriptomic techniques alone.

Post-translational modifications of transcription factors play key roles in transcription factor function. However, identification of these PTMs is challenging. Proteomics has played a significant role identifying key PTMs in this regard. Mass-spec analysis of immunoprecipitated RORγt from T_H_17 cells identified two key phosphorylation sites important for T_H_17 cell development; one phosphorylation site was critical for T_H_17 cell development whereas the other inhibited it (27). A separate study identified several acetylation sites on RORγt that became deacetylated by Sirtuin 1 (SIRT1), promoting autoimmunity (28). Quantitative phosphoproteomics has been used to characterize IL-23 signaling in T_H_17 cells (29). Data from these studies identified 168 phosphorylation sites occurring *via* IL-23 signaling leading to phosphorylation of the myosin regulatory light chain (pRLC-S20), a key regulator of actomyosin cytoskeleton contraction and cell migration. Their studies uncovered a key role for IL-23 in the regulation of cell motility. Alternatively, one group used high-resolution phosphoproteomic mass spectrometry to identify IL-2-mediated phosphorylation events in cytotoxic CD8^+^ T cells (CTLs) (30). IL-2 is critical for the clonal expansion of both CD4^+^ and CD8^+^ T cells, but prior to this study a global understanding of IL-2 signaling was lacking. Using phosphoproteomics, the authors provide a thorough characterization of the IL-2-regulated phosphoproteome, uncovering a SRC-family kinase-controlled phosphorylation network in CTLs and a separate coordinated phosphorylation cascade regulated by IL-2-JAK signaling. Specifically, they found that SRC kinases regulated a large component of the CTL phosphoproteome, controlling events distinct from other IL-2-JAK1/3-mediated events. SRC kinases were required to sustain the activity of critical metabolic kinases, including mTORC1 and AKT whereas IL-2-JAK signaling coordinated phosphorylation of transcription factors, chromatin regulators, mRNA translation, GTPases, etc. These data uncovered the complexity of protein phosphorylation in CTLs and established that future work identifying cellular phosphorylation kinetics is in order to determine how signaling pathways are coordinately regulated. Finally, biochemical and mass-spectrometric analysis of purified Foxp3 complexes from T regulatory cells (Tregs) revealed that Foxp3 exists in multiprotein, heterogenous complexes of 400-800kDa or larger (31). While over 350 associated proteins were identified, many were associated with transcription, including Cbfb, Bcl11b, Ikzf3, and Chd4. Interestingly, Foxp3 bound to and regulated a number of genes that serve as its own cofactors, including Runx, NFAT, and GATA-3. As GATA-3 was previously described to play a role in Treg function and homeostasis (32, 33), mass-spec analysis confirmed this regulatory role and further identified a subset of genes co-regulated by Foxp3 and GATA-3 important for Treg cell fate and function, including *Ets2*, *Satb1*, and *Klf3*. These studies are clear examples of how mass-spec based proteomics can identify key T cell regulatory events and aid in understanding of T cell biology.

Protein turnover in mammalian cells, including T cells, is regulated by many cellular processes, with mammalian target of rapamycin complex 1 (mTORC1), playing a significant role (34). Since a basic molecular understanding of its function at the protein level in T cells had yet to be established, one group used quantitative mass spectrometry to compare CD4^+^ vs. CD8^+^ T cell proteome restructuring upon antigen encounter to determine how mTORC1 regulated these events (1). Over 9,000 proteins were identified, demonstrating how environmental signaling pathways integrated with antigen and cytokine signaling to ensure specific and appropriate immune responses. The authors identified key differences between CD4^+^ and CD8^+^ T cells, including CD8^+^ T cells ten-fold increase in the amino acid transporter SLC1A5 relative to CD4^+^ T cells. They also found that CD8^+^ T cells also have higher concentrations of protein-translational machinery and nutrient transporters which could account for their enhanced proliferative capacity. While no major differences were observed with mTORC1 between CD4^+^ and CD8^+^ T cells, naïve CD4^+^ T cells have a unique dependence on mTORC1, particularly for cell-cycle regulation. This same group used a similar approach to better understand how the transcription factor, Myc, controls CD4^+^ and CD8^+^ T cell activation and metabolism following stimulation through the TCR (35). Data from this paper demonstrated Myc controls proteome restructuring in T cells, regulating amino acid transporters critical for T cell bioenergetic and biosynthetic programs the cell requires for proper growth and development. While both Myc-dependent and independent functions were uncovered in T cell metabolic processes, upregulation of amino acid transporters was part of a feed-forward loop sustaining Myc expression in T cells. These data shed light on why Myc is so important for specific T cell metabolic functions, including glutamine metabolism and glycolysis. To further pursue metabolic regulation in T cells, in a separate study this group used high-resolution mass-spec to map the proteome of CTLs to better understand mTORC1 and mTORC2 activity in CTL function (36). The authors mapped ~6800 proteins in CTLs and discovered that CTLs have high mTORC1 activity, playing a dominant role in CTLs over mTORC2. Despite this, only a small subset of the CTL proteome was controlled by mTORC1 activity; mTORC1 controlled expression of metabolic, effector, and adhesion molecules. When comparing the CTL transcriptome to the proteome, they found a discordance between mRNA and protein abundance. For example, while the mRNA expression of T-bet and Eomesodermin (EOMES) was comparable in CTLs, T-bet protein expression was significantly greater than EOMES. There was also a significance discordance between mRNA and protein expression of the IL-2 receptor. mRNA indicated a 3:1:2 stoichiometry of receptor chains (α:β:γ) whereas the corresponding ratio of protein was 92:1:2, respectively. Further, through use of mTORC1 and mTORC2 inhibitors, this study demonstrated the power of unbiased proteomics on understanding the effects of drugs in cells. Collectively, these studies highlight the power of quantitative untargeted analysis of protein content in understanding T cell function and metabolic regulation.

In contrast to the murine studies, human T cell proteomic studies have been a bit more confounding. For example, unlike mouse studies, there was strong correlation between proteomic and transcriptomic data depending on cell type and time points assessed. One group used a combination of proteomics and transcriptomics to study the effects of cytokines on human naïve (T_N_) and memory (T_M_) CD4^+^ T cell responses in an effort to better understand cytokine responses in memory cells, which to that point had not been studied in-depth (37). T_N_ and T_M_ cells were polarized towards four different CD4^+^ T helper subtypes (T_H_1, T_H_2, T_H_17, and iTreg), collected at 16h and 5 days post activation, and subjected to proteomic and transcriptomic analysis. Similar to the mouse studies, the authors found variation between the proteome and transcriptome during initial T cell activation. However, downstream of initial activation, there was high correlation between RNA and protein expression in all populations evaluated. One finding that was surprising was that while T_N_ could differentiate into any T helper population, T_M_ cells could not acquire a T_H_2 phenotype. Interestingly, T_M_ acquired a T_H_17 phenotype in response to iTreg signals, perhaps due to reliance on TGFβ signaling (38). When used in combination with transcriptomics, proteomics revealed a progression of T cells from naïve to central memory to effector memory T cell phenotypes. Progression was accompanied by increases in expression of effector molecules which enabled enhanced responsiveness to activation and cytokine signals from the environment.

In a separate study, evaluation of the proteome vs. transcriptome in human T_H_17 cells revealed a high correlation between datasets (39). However, when compared to a corresponding published mouse dataset (40), the authors found very little overlap between proteins expressed between human and mouse T_H_17 cells. This discrepancy could be due to the fact that the authors only compared their human T_H_17 data to one mouse data time point. Alternatively, activation conditions could also affect the overall proteome. A recent paper by Revu et al. demonstrated that anti-CD28 signaling is not required for human T_H_17 cell development *in vitro (* 41). Rather IL-23 signaling is sufficient as the second signal and these cells produce a more robust amount of IL-17A than traditional human T_H_17 *in vitro* differentiation conditions, amounts more consistent with mouse T_H_17 cells. In this particular study, it is unclear to what extent IL-17A was expressed in human T_H_17 cells relative to the ~45% IL-17A expressed in the mouse T_H_17 cells being compared to (40). Further, the authors saw little upregulation of key T_H_17 proteins, including IL-17A, IL-17F, and RORγt, in their proteomics data from human T_H_17 cells, suggesting the differentiation may not have been ideal. While it is important to perform parallel studies between mouse and human samples to understand the translational aspect of the work performed, when comparing datasets, particularly between two species, one should take caution when interpreting data. Cell types should not only be verified, their activation and differentiation states should be comparable for a fair and accurate comparison to occur.

There are other clear examples demonstrating differences between proteomic and transcriptomic datasets, which inevitably defined unique T cell subsets and signatures. One example stems from a study that set out to address how human Foxp3^+^ T regulatory (Tregs) cells resist acquisition into T effector or conventional cells. The authors used a combination of proteomics and transcriptomics on human blood derived naïve CD4^+^ T cells and effector Tregs (eTregs) and identified functionally distinct Foxp3^+^CD4^+^ T cells in human blood, defined a common Treg signature, and one specific for effector Tregs (eTregs) (42). These signatures comprised proteins involved in diverse cellular functions. The common Treg signature included Foxp3, Helios, metabolic proteins (GK, SHMT2), iron storage proteins, and lysosomal proteins. The eTreg signature included proteins involved in apoptosis, mitosis, DNA replication, etc. (42). Similar to what had been described with mouse samples, the proteomic Treg signatures defined had little overlap with transcriptomic signatures. The authors hypothesized the discrepancy may be due to the cellular state of the Tregs (i.e., steady state vs. activated) since stability of mRNA may be an issue (43). These differences may be less obvious in cycling cells where there is a need for protein synthesis. Regardless, the Treg signatures could not have been predicted based on transcriptomics alone. Collectively, these studies indicate the need to be careful with analysis and interpretation of proteomic data in T cells and consider cellular state and/or couple this approach with transcriptomic data to generate a full view of the T cell.

## Challenges with proteomics and future prospects: Single-cell mass spectrometry and beyond

While a powerful system, proteomics is not without its challenges keeping it from becoming a mainstream application. Unlike genomics and transcriptomics-based approaches, where one can sequence thousands of genes quickly, proteomics is not as high throughput. Additionally, the Core infrastructure for genomics and transcriptomics work is far more advanced than proteomics cores. For example, sample processing, instrumentation, and separation science involved with proteomics is not as mainstream as that which has been honed for Genomics. Data analysis is also technically demanding, requiring a level of expertise that are often only found in academic labs that perform proteomics on a routine basis. Thus, there is a significant need to develop pipelines for data analysis that is readily available to circumvent this issue. Perhaps one place to start would involve adapting pipelines currently used for transcriptomics towards proteomics.

Outside of these limitations, there are basic issues that hinder the mainstream use of proteomics. For example, unlike RNA and DNA, proteins can’t be amplified, so low abundance proteins, including transcription factors, are often missed. Another challenge is that data is sparse. In theory, all peptides should be “read” by the mass-spectrometer. However, this is not always the case and full coverage of the proteome is not acquired. Sample amount poses another obstacle for making proteomics more mainstream. The advent of single-cell RNA-sequencing (scRNA-seq), Assay for Transposase-Accessible Chromatin (ATAC)-seq and CUT&RUN has enabled the interrogation of small numbers of cells to address biological questions. These assays require low cell numbers and are particularly useful when trying to determine how certain cell-types drive disease. However, the number of cells currently needed for proteomics far outpaces that of assays like scRNA-seq, limiting our ability to interrogate *ex vivo* isolated T cells from mouse models of disease. Finally, while methodology for phosphoproteomics has significantly advanced, there are multiple types of PTMs which regulate cellular function, including sumoylation and glycosylation. Further, a deeper understanding of the subcellular localization of PTMs, down to the organelle level, is needed to better understand molecular signaling events in cells. While several methods have been developed in an attempt to do this, including enzyme proximity labeling methods like APEX/APEX2 (44, 45) and BioID/TurboID (46–48), they are not without their caveats. APEX, which utilizes hydrogen peroxide can affect cellular metabolism and consequently, PTM status (49). TurboID and similar methods are subject to pervasive biotinylation of endogenous proteins, not only generating an over estimation of PTMs in the cell but may also lead to cell toxicity and perturbations in normal cellular responses (50). Recent advances using uncaging-assisted biotinylation and mapping of phosphoproteome (SubMAPP) (51) may help alleviate some of these promiscuity and toxicity issues. However, it is clear that we need better methodology to interrogate PTMs, both globally and at the organelle level, to gain a better understanding of how they affect T cell function.

While measuring changes in protein abundance is of critical importance to understand cell fate decisions, structural remodeling of proteins is equally so. Several methods exist to measure protein structure at the atomic level, but few methods can directly observe protein structure in functioning cells. Recent developments have started to tackle this issue. The release of AlphaFold, an artificial intelligence (AI) system that predicts the 3-dimensional structure of proteins based off its 1-dimensional amino acid sequence, has significantly advanced our understanding of static protein structure (52). However, there are still some limitations to this software (i.e., disordered protein regions cannot be accurately resolved), the proposed structures still need validation, and proteins are not static, rather always in motion. However, this software can predict biological insights and fuel hypothesis driven research into protein function and interactions in live cells that can be tested experimentally. Use of approaches like limited proteolysis coupled to mass-spectrometry (LiP-MS), which identifies protein structural changes in their biological context on a proteome-wide scale, is a step above AlphaFold, enabling observations of structural changes. The idea behind this approach is that residues in a protein structure may be shielded from proteolytic digestion by PTMs, interactions with other molecules, or other parts of the protein. Using timed proteolytic digestion, which results in peptides that reflect protein/structure accessibility, coupled with mass-spec, one can predict protein structure in a complex biological setting (53, 54). These approaches may potentially be used to complement protein abundance readouts in order to maximize detection of alterations of cellular biological states. Regardless, advances in methods sensitivity and data analysis pipelines, similar to the deep machine learning algorithms developed for genomics-based research, is required to advance structural proteomics and uncover significant insights into functionality, helping uncover “hidden” information in the peptide/peptide-fragment data that comes off the mass-spectrometer.

**Figure 1:**
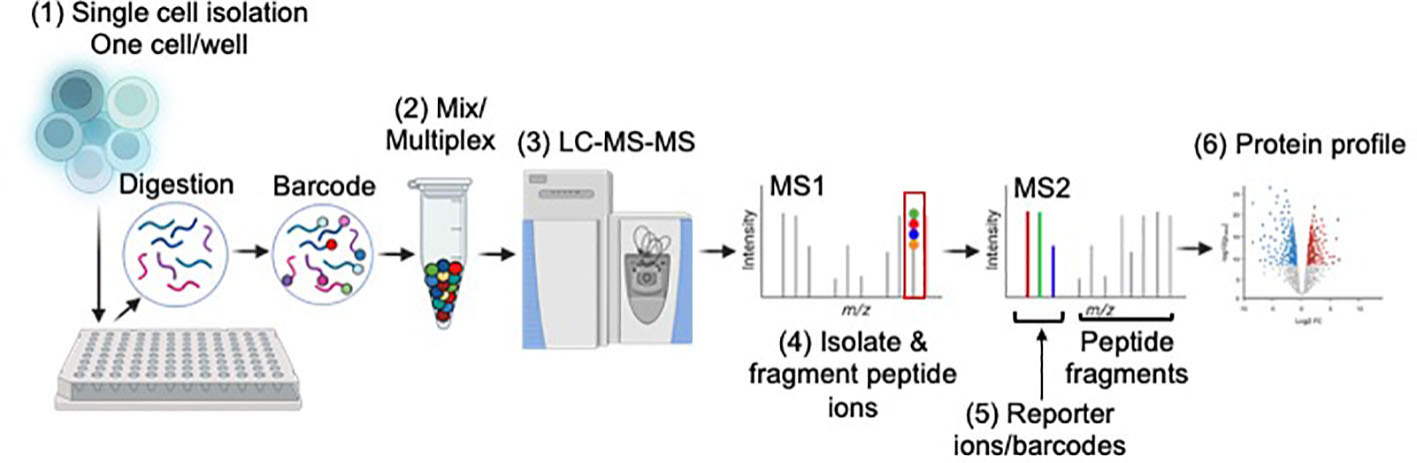
Basic overview of single-cell mass spectrometry workflow. Single-cell proteomics tends to use a “bottoms-up” approach. (1) Single cells are sorted into multi-well plates (one cell/well), lysed and digested via trypsin. Peptides in each well are labelled with barcodes, or tags, so researchers can determine how much of a given protein is present in each cell. One commonly used tag, tandem mass tag (TMT) can differentiate up to 18 samples in a single mixture. (2) TMT requires samples to be labeled individually then mixed for multiplexing. (3) Mixed samples are injected into the LC-MS. (4) Individual peptide species are ionized, isolated, and fragmented. (5) Analysis of the peptide fragments and barcodes allows for peptide sequence and protein quantification at the single-cell level, respectively. (6) Proteome profiling revealing protein expression of single cells is obtained. Image developed using BioRender.

While still in its infancy, recent advances in instrumentation and methodologies has enabled the measure of lipids, proteins, and small molecules in a single cell using mass spectrometry (55). (**Table 1**; **Figure 1**) The implementation of microfluidic chip systems, facilitating reactions to occur in a small volume, has greatly accelerated this approach. ProteoCHIP and nanodroplet processing in one pot for trace samples (nanoPOTS) are two such designs (61, 68). For example, nanoPOTS uses a chip-based nanodroplet processing platform for enhancing proteomics processing and sample analysis for small cell populations. nanoPOTS reduces the processing volumes (<200nL, down from hundreds of microliters), while maintaining parameters deemed essential for optimal bulk proteomic sample preparation. Reduced processing sample volume minimized absorptive loss of the sample, which had previously been the bottleneck of sample loss during preparation. Using nanoPOTS, the group was able to achieve reproducible coverage of over 3000 proteins from approximately 10-100 cells or clinical samples. Specifically, they were able to quantify ~2400 proteins from single human pancreatic islet cross-sections, isolated by laser microdissection, from a patient with type 1 diabetes vs a healthy control (61). The authors indicated that their platform could interface with cell sorting *via* flow cytometry, which would be an ideal workflow for immunologists wishing to perform similar experiments. Optimizing on this platform, another group developed an “all-in-one” proteomic sample preparation and data acquisition for single-cell proteomics called SciProChIP-DIA. This method multiplexes samples, automates cell isolation, preparation, cell counting, imaging, and sample processing coupled with a data-independent acquisition mass-spectrometry approach in a single device. Essentially, they developed a fully automated workflow for low-input samples that minimizes sample loss while achieving high reproducibility and sensitivity. Using this automated approach, researchers were able to characterize up to 1,500 proteins per cell, with a false discovery rate of 1% in both human adenocarcinoma cells (PC-9) and chronic B cell leukemia cells (MEC-1) (69). Compared to other studies using single-cell proteomic profiling, SciProChIP-DIA demonstrated more sensitive coverage than others tested, including nanoPOTS. The authors postulated that SciProChIP-DIA was versatile and scalable in order to fit various applications, including the study dynamic proteomic alterations upon cell stimulation.

**Table 1:** Recent Single-cell Proteomic Studies.

MS Approach	Number of proteins identified	Cell type	References
nanoPOTS	731	MCF10A	(56)
nanoPOTS	874	HeLa	(57)
nanoPOTS	1056	HeLa	(58)
nanoPOTS	~1600	Mouse epithelial, endothelia, immune cell lines	(59)
nanoPOTS	~700	FM1-43	(60)
nanoPOTS	~1500-3000	HeLa, human islet sections	(61)
nanoPOTS	~1600	Rat brain sections	(62)
SCOPE-MS	767	Jurkat, U-937	(63)
SCoPE2	~3000	U-937, HEK293	(64)
FUNpro/SCOPE-MS	>1000	U20S	(65)
DVP	>500	U20S	(66)
T-SCP	>550	HeLa	(67)

Recent advances from the Mann lab have pushed the boundaries of single-cell mass spec. In a recent publication, they highlight a new technique called Deep Visual Proteomics (DVP) (66). This method combines imaging technologies with unbiased proteomics to quantify the number of expressed proteins in a cell, map tissue or cell-type specific proteomes, or to identify drug targets in a cell. Using this approach, they uncovered cellular phenotypes and dysregulated pathways with automated single cell laser microdissection coupled with ultra-high sensitivity mass-spec in melanoma progression. This system is applicable to any biological system that can be imaged and similar to another application recently described, called FUNpro (65). FUNpro also uses a microscopy-based method, coupled with SCOPE-MS, to perform functional single-cell proteomic-profiling in order to link the proteome to cellular phenotype. Both methods can identify and characterize rare cell states and interactions. The Mann lab has also recently developed a workflow that enables higher sensitivity in single cell proteomics. Using FACS-isolated cells, they injected single cells one by one into the mass-spec, which they call true single-cell-derived proteomics (T-SCP) (67). Using their improved automated workflow, they demonstrated increased sensitivity up to two orders of magnitude greater than previous methods and were able to dissect arrested cell cycle states in cancer cells (HeLa cells). These works have increased the sensitivity of scMS. Unfortunately, most of these studies have been performed using cell lines or cells isolated from fixed tissue. Future work will need to entail single cell proteomics of primary cells, including T cells, from freshly isolated ex-vivo samples (i.e., murine or PBMCs) to generate the most robust visualization of the proteomics landscape *in vivo*.

Given the sensitivity between approaches, single-cell proteomics tends to use a ‘bottom-up’ approach to identify proteins rather than looking for intact proteins. (**Figure 1**) Therefore, key challenges for improvement are similar to those described above, including analyte coverage and depth of analyte characterization. Regardless, advances in single-cell mass-spec has rapidly improved over the last 5 years (55). Should this rate continue, one can envision use of this technique on a more routine basis to address questions to better understand T cell heterogeneity and exhaustion in tumors. This knowledge could identify novel therapeutic approaches for immune-oncology. Single-cell mass-spec could also be used to gain a better understanding of events dysregulated at the protein level in autoreactive T cells – events that are not likely captured by transcriptomics alone. Like cancer therapeutics, this information could lead to potential novel therapeutic options for various autoimmune diseases. Regardless, use of proteomics, whether bulk or at the single cell level, is imperative to characterize the molecular signatures of normal vs aberrantly activated T cells, information that is currently lacking but clearly needed to further advance our understanding of the mechanisms of biology and disease.

## Author contributions

LS gathered all relevant data and articles, wrote, edited, and revised the manuscript.

## Funding

This work was supported by the National Institutes of Health grant numbers R21AI164722 and R01CA241816.

## Conflict of interest

The author declares that the research was conducted in the absence of any commercial or financial relationships that could be construed as a potential conflict of interest.

## Publisher’s note

All claims expressed in this article are solely those of the authors and do not necessarily represent those of their affiliated organizations, or those of the publisher, the editors and the reviewers. Any product that may be evaluated in this article, or claim that may be made by its manufacturer, is not guaranteed or endorsed by the publisher.
